# Impacts of stimulus parameters and configurations on motor cortex direct electrical stimulation using intrinsic optical imaging: a pilot study

**DOI:** 10.1186/s12938-022-01026-2

**Published:** 2022-08-29

**Authors:** Long Liu, Jiacheng Zhang, Jie Sun, Kedi Xu

**Affiliations:** 1 Department of Neurosurgery, Ningbo City First Hospital, No.59 Liuting Street, Ningbo, 315010 Zhejiang China; 2 Zhejiang Provincial Key Laboratory of Cardio-Cerebral Vascular Detection Technology and Medicinal Effectiveness Appraisal, Key Laboratory of Biomedical Engineering of Education Ministry, Department of Biomedical Engineering, Zhejiang University, Room 511, Zhou Yiqing Building, 38 Zheda Road, Hangzhou, 310027 China; 3 Qiushi Academy for Advanced Studies (QAAS), Zhejiang University, Hangzhou, 310027 China; 4 Zhejiang Lab, Hangzhou, 311100 China

**Keywords:** Cortical stimulation, Monopolar, Bipolar, Multi-electrode configurations, Interleaved stimulation

## Abstract

**Background:**

Motor cortex stimulation applied as a clinical treatment for neuropathic disorders for decades. With stimulation electrodes placed directly on the cortical surface, this neuromodulation method provides higher spatial resolution than other non-invasive therapies. Yet, the therapeutic effects reported were not in conformity with different syndromes. One of the main issues is that the stimulation parameters are always determined by clinical experience. The lack of understanding about how the stimulation current propagates in the cortex and various stimulation parameters and configurations obstruct the development of this method.

**Methods:**

In this study, we investigated the effect of different stimulation configurations on cortical responses to motor cortical stimulations using intrinsic optical imaging.

**Results:**

Our results showed that the cortical activation of electrical stimulation is not only related to the current density but also related to the propagation distance. Besides, stimulation configurations also affect the propagation of the stimulation current.

**Conclusions:**

All these results provide preliminary experimental evidence for parameter and electrode configuration optimizations.

## Background

Electrical stimulation on the central nervous system such as deep brain stimulation (DBS) has been considered a promising treatment to alleviate symptoms of neurological disorders including Parkinson’s disease, essential tremor, chronic pain, depression, post-stroke pain, and epilepsy [1, 2]. However, DBS is not suitable for all symptoms or situations. In parallel with the development of DBS, cortical stimulations are also available as treatments for neurological and psychiatric disorders.

Noninvasive methods such as transcranial magnetic stimulation (TMS) [3] and transcranial direct current stimulation (tDCS) [4] can activate neural structures without surgical procedures, being applied in recovery from brain injuries and modulation of cortical activities recently [5–8]. Yet, the treatment results reported by clinical studies are not consistent, which may partially be caused by variations in stimulation parameters, such as stimulation location, intensity, and frequency used [9]. While further large-scale studies are necessary for systematic study before stimulation parameter optimization, one of the main limitations that cannot ignore is less focused stimulation due to penetrating the scalp [5].

Cortical stimulation with implanted electrodes also shows its appealing application as a neurosurgical method for neural modulation intervention therapies [10]. Due to direct contact with the brain tissue without a skull, this approach delivers relatively stronger stimulations to a more focused target than noninvasive stimulation [11, 12], providing an alternative application for both chronic neurological disorder treatment and sensory information feedback.

Motor cortex stimulation has been used as a therapeutic tool for neuropathic disorders since its introduction in 1991, including relieving neuropathic pain [13], treatment of epilepsy [14], tremor, and Parkinson’s disease [10], supporting the post-stroke recovery of motor functions [15], as well as providing sensory feedbacks in brain–machine interface (BMI) studies [16, 17]. However, the mechanism and effectiveness are still not fully understood. Clinical studies have shown that different syndromes may respond differently to neuromodulation. C. Michael Honey et al. [18] have reviewed the literature and found consistent effectiveness for deafferentation facial pain and post-stroke pain rather than lower limb pain. One of the main issues is that the stimulation parameters are always determined primarily by clinical experience. The lack of understanding about how stimulation current propagates in the cortex and various stimulation parameters and configurations obstruct the development of this method.

The efficacy of cortical stimulation mainly depends on the frequency, intensity, and polarity of the stimulation [19]. The intensity of stimulation may influence the distribution of current density [20] and electric field. Additional factors include electrode configurations [21] and the distance between the electrodes [22]. Different electrode configurations produce different shapes and distributions of electrode fields [23]. The monopolar electrode configuration uses only negative contact (cathode) for stimulation in the targeted area, with the positive contact (anode) far away on the occipital bone. A bipolar electrode configuration utilizes a pair of electrode contacts within its stimulation structure, one acting as negative contact and the other a positive one. A computational study has shown that in this case, the neurons respond differently from a simple linear combination of responses to the same two monopolar stimulation referenced to a distant ground [24], producing more complex activation zones [25]. Furthermore, a multi-electrode configuration having one current source and several surrounding return electrodes is believed to decrease the amount of current leakage to surrounding areas [26], suggesting specificity improvement with this multi-electrode configuration [24]. These computational studies reveal the current density distribution of different electrode configurations, which are relevant to the prediction of therapeutic efficacy. However, there is still a lack of experimental studies examining the theoretical conclusions, since the selection of a proper brain model may also influence the validity of results.

On the other hand, safety is always the most important concern in electrical stimulation therapies. An optimal approach should be effective in activating targeted neural structures without bringing damage to brain tissues. Therefore, stimulation parameters should be carefully considered. Recent studies have reported interleaved stimulation (ILS) as an alternative to conventional DBS with monopolar or bipolar stimulation [27]. By delivering two sets of stimulation in an alternating sequence, interleaved stimulation has the advantage of alleviating stimulation-induced side effects while individually optimizing specific symptoms of different brain regions [28]. So far, interleaved stimulations have been applied mainly in DBS therapeutics, and few studies have focused on the effects of ILS with cortical stimulation. Considering the performances observed with ILS DBS, it may be a promising configuration for clinical practice.

Optical imaging of intrinsic signals is an optical imaging method that is based on changes in optical properties of neuronal tissue caused by physiological activities [29]. At a wavelength of 630 nm, intrinsic signals reflect local blood oxygenation changes, which arise from changes in neuronal activity due to neurovascular coupling [30, 31]. This technique allows in vivo functional imaging with a high spatial resolution with relatively simplistic experimental setups [32]. In this study, we applied cortical stimulation through an ECoG array while monitoring tissue reflectance from M1. Our goal was to preliminarily investigate the effect of different stimulation configurations on neural activation produced by cortical stimulations. Quantitative analyses of response intensity and distribution range on the cortex were conducted, to evaluate the influence of different stimulation configurations and parameters.

## Results

### Comparison between monopolar and bipolar cortical stimulation

**Fig. 1:**
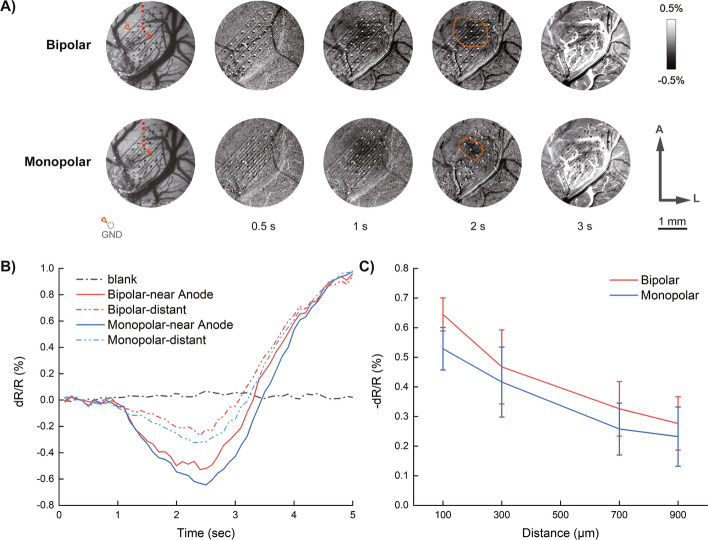
Cortical responses to bipolar and monopolar stimulations. (** A**) Optical responses to bipolar (*top line*) and monopolar (*bottom line*) stimulations over time. Vessel maps obtained with green light were shown on the *far left*. Electrodes used were illustrated with *solid* (sources) or *hollow* (returns) arrows. Subtracted frames shown were obtained at 0.5 s, 1.0 s, 2.0 s, 3.0 s, from imaging onset, respectively. Electrical stimulations were conducted between 0.5 s and 1.0 s during imaging. Areas of the image having reflectance changes larger than 3 times the mean square deviation were labeled with red lines as areas with significant changes. A: anterior; L: lateral. (**B**) Super pixel time courses of bipolar and monopolar stimulations. The “near” courses were obtained from the red dots nearest to source electrodes indicated in (**A**) and the "distant" ones were obtained from the far-most dots in (**A**). (**C**) Peaking values of time courses as function of distance from the “near” dots to the “distant” dots indicated in (**A**)

Both monopolar and bipolar stimulations were performed through an ECoG electrode array under optical imaging. Figure 1 demonstrates typical reflectance changes evoked by the two stimulation methods. As expected, both stimulations evoked consistent reflectance trends over time, reaching their peaks at about 1.5 s after stimulation onset. Meanwhile, the time courses show larger peaking values evoked by bipolar stimulation than monopolar did, with the same stimulation parameters. The areas near electrode contacts (red dots near the solid arrows in Fig. 1A) show significant differences in reflectance changes. In addition ROI calculations from distal aretohowed no statistical differences between these two stimulation methods. The calculated results of ASC show a similar trend with that of MRC, with larger response areas evoked by bipolar stimulation than monopolar.

**Fig. 2:**
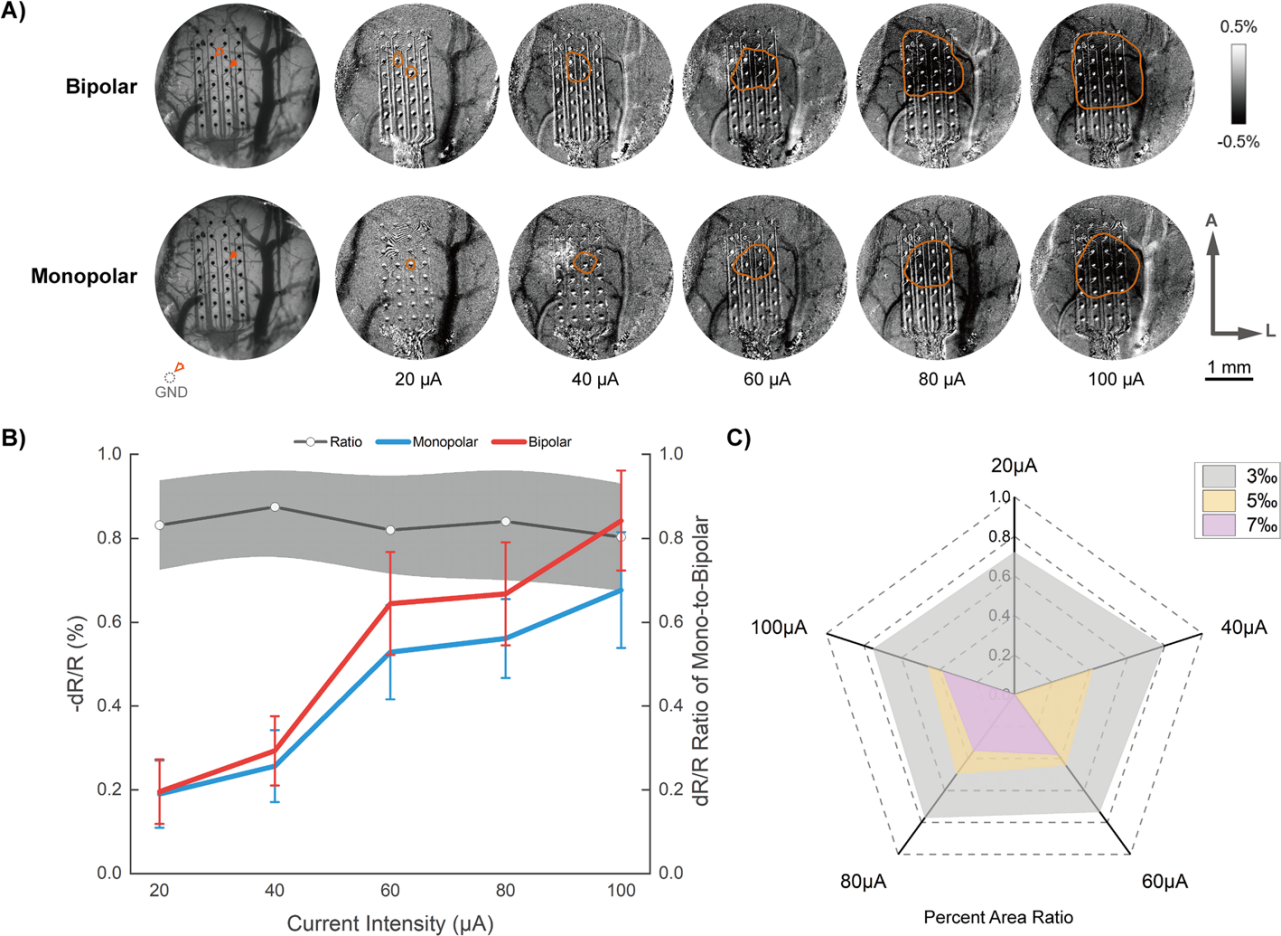
Effects of current intensity modulation. (**A**) Typical optical responses to bipolar (*top line*) and monopolar (*bottom line*) stimulations with different current intensities. Vessel maps obtained with green light were shown on the *far left*. Significant changing areas were labeled with red lines. A: anterior; L: lateral. (**B**) Maximum reflectance changes to current intensity changes among all rats imaged. The cortical responses showed a near-linear relationship with amplitude changes. The black line with gray shadows indicates the calculated changing ratios of monopolar to bipolar stimulations. (**C**) Calculated changing area ratios of momopolar to bipolar stimulations. The pixels of the areas having more than 3‰, 5‰, 7‰ reflectance changes were calculated respectively

We then measured the effects of current intensity and frequency changes. As illustrated in Fig. 2, the two stimulation methods show similar trends in response to current intensity changes, with constant increases in MRCs resulting from increasing current intensity. Repeated-measures ANOVA reveals significant effects of both monopolar vs bipolar (*p* < 0.0001, *F* = 184.36), and current intensity (*p* < 0.0001, *F* = 194.56) in current modulation tests. Frequency modulation also shows significant effects (*p* < 0.001, *F* = 54.69). Post hoc testing with Bonferroni’s multiple comparisons shows that monopolar stimulations and bipolar stimulations differed significantly (*p* < 0.001). For current intensity, stimulation with 20 to 80 μA all show significant dereferences from each other, while stimulation with 80 μA and 100 μA shows no significant difference from each other. For frequency changes, stimulation at 20 Hz differed significantly from that at 10, 50, 100, 200 Hz, while others did not differ significantly from each other. To further discover whether the changing trends were consistent between the two stimulation methods, we calculated the changing ratios of monopolar to bipolar stimulations. The analytical results show that the ratios of intensity modulation remain within a stable range with no significant difference found, suggesting that the effect of current intensity modulation is not affected by the conditions of the electrode connections.

As to spatial distribution, we calculated the pixels of the areas having more than 3‰, 5‰, 7‰ reflectance changes (3‰, 5‰, 7‰ ARC for short, respectively) in each subject. For demonstrations of the differences between the two stimulation methods, we also calculated the relative changing ratios of monopolar to bipolar results. Figure 2C demonstrates the calculated results of changing area ratios. Compared to monopolar stimulations, bipolar stimulations resulted in larger changing areas in all the conditions we tested. Besides, monopolar stimulations with lower current intensities (20 and 40 μA) could hardly evoke strong reflectance changes (5‰ and 7‰ ARCs), and the area ratios also showed quick drops with larger reflectance changes, indicating that a threshold is required for activating brain activities to a certain extent. These results demonstrate a stronger and more focused reflectance effect evoked by bipolar stimulation. Notably, although the reflectance changes of monopolar stimulation seemed to be lower than that of bipolar stimulation, once reaching the threshold, the area ratio remained relatively stable with the increase of stimulation intensities.

**Fig. 3:**
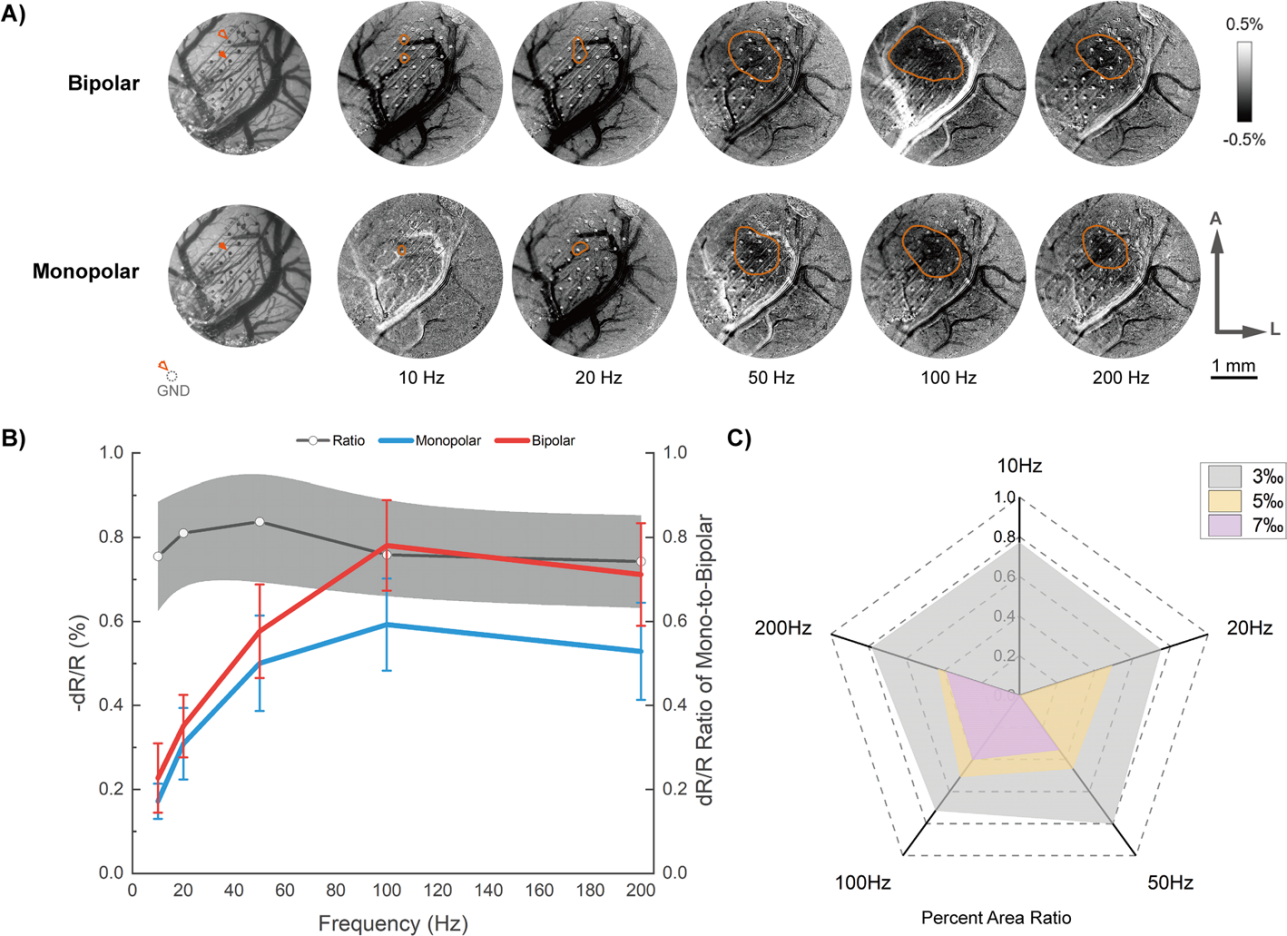
Effects of frequency modulation. (**A**) Typical optical responses to bipolar (*top line*) and monopolar (*bottom line*) stimulations with different frequencies. Vessel maps obtained with green light were shown on the *far left*. Significant changing areas were labeled with red lines. A: anterior; L: lateral. (**B**) Maximum reflectance changes to frequency changes among all rats imaged. The cortical responses showed peaking changes at 100 Hz. The black line with gray shadows indicates the calculated changing ratios of monopolar to bipolar stimulations. (**C**) Calculated changing area ratios of momopolar to bipolar stimulations. The pixels of the areas having more than 3‰, 5‰, 7‰ reflectance changes were calculated respectively

Frequency modulation leads to different situations. As shown in Fig. 3, changes in frequency result in non-linear changes of reflectance, with peaking changes appearing around 100 Hz. However, the changing ratios of the two stimulations remain stable throughout the frequency changes, and no significant changes were found. As to changing areas, similar results were found with intensity changing conditions. Calculated results of ASC reveal similar trends to reflectance peaking values, whereas bipolar stimulation evoked a larger response of 5‰ and 7‰ ARC. These results again suggest a relatively more concentrated activation area with bipolar stimulation than monopolar stimulation.

### Impacts of electrode spacing on cortical stimulation

**Fig. 4:**
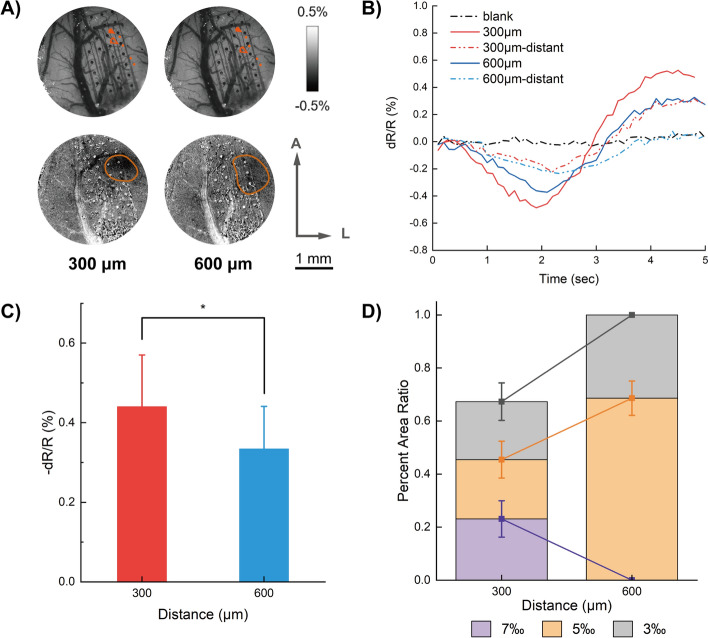
Impacts of electrode spacing. (**A**) Illustrations of electrode configurations with vessel maps (*top line*) and corresponding subtracted changing areas calculated from stimulations with different electrode spacings (*bottom line*). A: anterior; L: lateral. (**B**) Super pixel time courses of the two conditions. The solid lines were obtained from the red dots nearest to source electrodes indicated in (**A**) and the hollow ones (labeled “*distant*”) were obtained from the far-most dots in (**A**). (**C**) Maximum reflectance changes among all rats imaged. The two different electrode spacing conditions showed significant differences. *:* p* < 0.01. (**D**) Calculated changing area ratios of the two conditions. The pixels of the areas having more than 3‰, 5‰, 7‰ reflectance changes were counted, and the ratios were then calculated to the maximum value (3‰ at 600 μm electrode distance), respectively

Next, we measured the influences of electrode spacing. Two different spacing conditions were tested: two neighboring electrode contacts, and a doubled spacing pair of electrode contacts (spacing of 300 μm, and 600 μm, respectively). Reflectance changes show larger peaking values in response to the shorter spacing pairs of electrodes (300 μm), with significant differences between the two conditions (repeated measures ANOVA, *p* < 0.01, *F* = 44.92; Bonferroni’s multiple comparison, *p* < 0.01, Fig. 4B, C). Whereas significant changing areas show larger spread areas with the longer spacing condition (600 μm). Advanced analyses indicate that although larger electrode spacing enlarges spread areas, the area with 5‰ or higher reflectance changes were hardly found in large spacing conditions (Fig. 4D), suggesting that the activation of electrical stimulation is not only related to the current density but also related to the propagation distance. Comparatively, under short electrode spacing conditions, cortical responses reveal a more focused and balanced spread among the affected area (repeated measures ANOVA, *p* < 0.0001, *F* = 573.28; Bonferroni’s multiple comparison, *p* < 0.01). These results indicate that spatial specificity effects of electrical stimulation could be adjusted by the spacing of electrodes to some extent, the smaller the spacing, the stronger and more concentrated the response is.

### Effects of multi-electrode stimulation

**Fig. 5:**
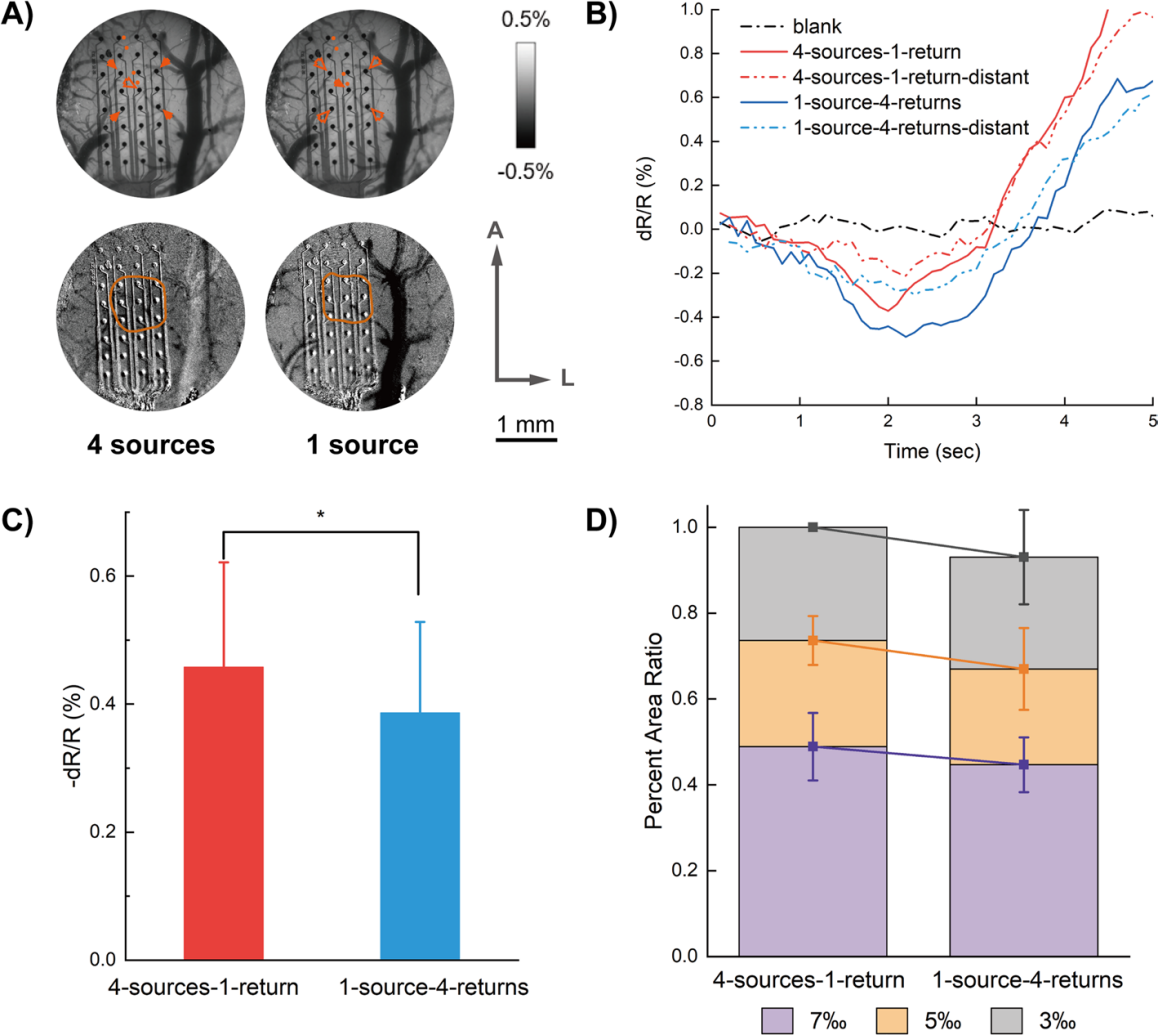
Effects of multi-channel configurations. (**A**) Illustrations of electrode configurations with vessel maps (*top line*) and corresponding subtracted changing areas calculated from stimulations with different configurations (*bottom line*). A: anterior; L: lateral. (**B**) Super pixel time courses of the two conditions. The solid lines were obtained from the red dots nearest to central electrodes indicated in (**A**) and the hollow ones (labeled "distant") were obtained from the far-most dots in (**A**). (**C**) Maximum reflectance changes among all rats imaged. The two different configurations showed significant differences. *:* p* < 0.01. (**D**) Calculated changing area ratios of the two conditions. The pixels of the areas having more than 3‰, 5‰, 7‰ reflectance changes were counted, and the ratios were then calculated to the maximum value (3‰ at 4 sources-1 return arrangement), respectively

Researches have shown that properly patterned multi-electrode simultaneous stimulations may produce electrical fields with desirable spatial specificity. In the current study, quadrilateral configurations of the cathodic–pulse stimulation were applied in this part of the study. In 4 sources-1 return configuration, 4 electrode contacts delivered cathodic pulses simultaneously, and the central electrode was used as the return electrode. In 1 source-4 return arrangement, the centered electrode acted as a single source and the surrounding 4 electrodes were used as return electrodes. To eliminate deviation induced by total charge differences, current intensity applied in 4 sources-1 return arrangement was a quarter the intensity delivered to 1 source-4 returns condition. Imaging results show that reflectance responses are significantly stronger with 4 sources-1 return rather than 1 source-4 return arrangement (repeated measures ANOVA, *p* < 0.0001, *F *= 144.31; Bonferroni’s multiple comparison, *p* < 0.01, Fig. 5B, C. Response areas also show a significant difference between the two conditions, revealing a larger spread with 4 sources-1 return arrangement (repeated measures ANOVA, *p* < 0.0001, *F* = 412.74; Bonferroni’s multiple comparison, *p* < 0.01, Fig. 5D). These results indicate that multi-electrode simultaneous stimulations could modulate spatial spreading specifically.

### Comparison between simultaneous and interleaved stimulations

**Fig. 6:**
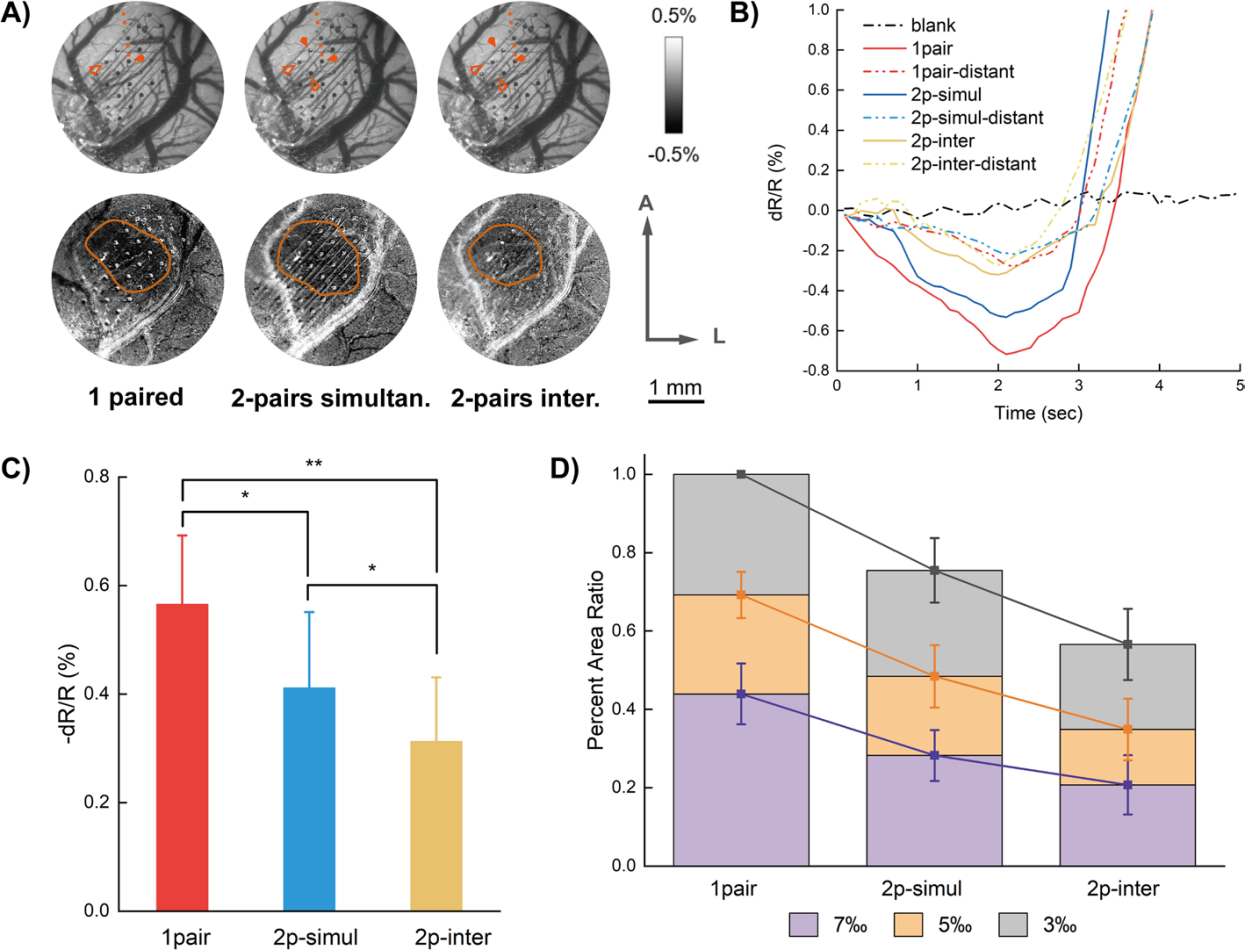
Responses to interleaved stimulation. (**A**) Illustrations of electrode configurations with vessel maps (*top line*) and corresponding subtracted changing areas calculated from stimulations with different conditions (*bottom line*). A: anterior; L: lateral. (**B**) Super pixel time courses of the different conditions. The solid lines were obtained from the red dots nearest to source electrodes indicated in (**A**) and the hollow ones (labeled “distant”) were obtained from the far-most dots in (**A**). (**C**) Maximum reflectance changes among all rats imaged. The three different conditions showed significant differences between each other. *:* p* < 0.01; ** p < 0.001. (**D**) Calculated changing area ratios of the different conditions. The pixels of the areas having more than 3‰, 5‰, 7‰ reflectance changes were counted, and the ratios were then calculated to the maximum value (3‰ at one pair electrode stimulation condition), respectively

Another stimulation condition recommended is interleaved stimulation. Interleaved stimulation with two pairs of electrodes could reduce charge accumulation and fatigue effect, and is considered safe for long-term stimulation. We compared responses to stimulations with one pair of electrodes, two pairs of electrodes simultaneously, and two pairs of electrodes interleaved. To ensure an equal total electric quantity delivery, one-pair stimulations were applied with 10 pulses, and two-pairs stimulations were applied with 5 pulses. Reflectance changes show significant differences among all three conditions, with the largest responses evoked by one single pair of electrodes, and the smallest responses responded to interleaved stimulations (Fig. 6B, C). Repeated measures ANOVA shows significant effects among the three conditions (*p* < 0.0001, *F* = 439.69); and Bonferroni’s multiple comparison reveals significant difference between one pair of electrode and two-paired interleaved stimulations (*p* < 0.01). As to response areas, the single-pair-electrode stimulation also showed the largest affected area, while the two-paired interleaved stimulation resulted in the smallest changing area. This may be due to the different stimulation durations (pulse numbers) of the different conditions. Repeated measures ANOVA shows significant effects among the three conditions (*p* < 0.0001, *F* = 655.80); and Bonferroni’s multiple comparison reveals more significant differences between one pair of electrode and two-paired simultaneous stimulations and between one pair of electrode and two-paired interleaved stimulations (*p* < 0.001), the two conditions of two-paired stimulations also show significant difference between each other (*p* < 0.01).These results show that, although the response intensity reduced, the interleaved stimulation remains relatively even spatial effects.

## Discussion

Cortical stimulation is showing its promising applications in several fields, but different research or clinical groups use various stimulation parameters and thus resulting in inconsistent effects. To improve the understanding of the parametric influences of electrical stimulation on neuronal activities, in the current study, we conducted electrical stimulation experiments on the rat motor cortex for both qualitative and quantitative analyses, and some preliminary results were obtained in agree with previous theories and studies.

Optical imaging of intrinsic signals is an optical method to study the functional architecture of cortex indirectly by measuring changes in blood oxygenation and optical properties of neural tissue [33]. Under the illuminating light of 625 nm, the reflectance signal is dominated by changes in the oxygenated hemoglobin [34], presenting the same physiological events as the “initial dip” obtained during BOLD fMRI [35]. Though the temporal time course shows a much slower change than that of neural activities, the initial negative dip is relevant to integrated synaptic activity [36, 37]. Takashima et al. have compared intrinsic signal optical imaging (OI) with voltage-sensitive dye imaging (VSD), they have found that the initial period of oxygenated hemoglobin change signal in OI was similar to the VSD map in the extent of the area of depolarizing neural activity [38]. Since the oxygenation change is preceded by the signal of cerebral blood volume (CBV) change, the OI signal during the initial period may reflect the oxygen consumption before CBV changes occur in the cortex, which occupies approximately the same cortical area as the depolarizing neural activity. Since OI has a high spatial resolution but relatively low temporal resolution, in this study, we took local response intensity and response diffusion range as comparison criteria.

To understand the cortical responses to cortical stimulations with different parameters and configurations, we compared the influences of current intensity and frequency changes in both monopolar and bipolar stimulation conditions. Besides, the effects of several commonly applied stimulation configurations were also estimated, including inter-electrode distance, electrode array arrangement, and stimulation parameters. Our optical imaging results show relatively large individual differences. Considering the anatomic differences, such as dura thickness, cerebrospinal fluid conditions will affect the spread of stimulation as well as distribution of electric field [39], in this study, we normalized the data within each rat. The normalized results show good consistency of response changes to parameters, indicating the reliability of our normalization process.

Modulation of current intensity results in near-linear responses on the targeted cortex. The previous study has shown that within 75% movement-inducing current (MIC), increasing the amplitude of stimulation caused a significant increase in neuron firing rates in the upper layers of the primary motor cortex with cathodic-first pulses [40]. It is hypothesized that with the amplitude of the electric field applied, neuronal compartments could polarize linearly [41]. Our imaging results support these previous studies in both bipolar and monopolar stimulation conditions. As to frequency modulation, the cortical responses showed frequency specificity. Stimulations with frequencies higher than 100 Hz did not evoke larger reflectance changes or spatial distributions. The frequency dependence was supported by previous electrophysiological and imaging studies [42–45], suggesting that peak benefit frequency should be carefully selected for functional stimulations.

For scientific research and clinical utilization of cortical stimulation, some groups use a pair of neighboring electrodes as current loops (bipolar), while others place the return electrode at the distant end (monopolar). Thus, discovering the difference of spatial diffusion of stimulation effects under the two electrode configurations may provide some evidence for subsequent chooses of appropriate configuration. Our imaging results indicate that under the same stimulation parameters, the local response intensities evoked by monopolar stimulation are lower than that of bipolar stimulation. However, further analysis of the response ratios from monopolar stimulation to bipolar stimulation reveal consistent values around 0.8 through all the parameters tested, with no significant differences found, indicating that this property is independent of the placement of the return electrode.

As to spread areas, with the increase of the stimulus intensity, diffusion areas with significant reflectance changes enlarged in both monopolar and bipolar stimulation conditions, with a relatively stable ratio of around 0.8. On the other hand, the calculated result of 5‰, and 7‰ ARC evoked by monopolar stimulation were significantly lower than bipolar conditions. One possible reason is that the distribution of electrode field is more concentrated in bipolar stimulation conditions. Computational studies have shown that for monopolar stimulation, the current density field is circular, while with bipolar stimulation, the current density field is more confined toward the axis of the bipolar [46]. Considering the electrode sizes and distances between electrodes in our experiment, the electric field between the two electrodes in bipolar condition may be overlapped, resulting in a stronger outcome than monopolar stimulation.

Simulation studies have shown that the diffusion of the electric field can be effectively controlled by changing electrical configurations. Our results also support this hypothesis. Varying electrode spacing can significantly change local response intensity as well as diffusion areas, with the trend of diffusion area changes showing a more complex relationship than linear or quadratic decreases with the increase of electrode spacing. Some researchers have proposed the use of a multi-electrode array to control current leakage and to ensure the uniformity of electric field [26, 47]. These studies are mainly focused on deep brain stimulations and neural prosthesis studies. Our results showed that with motor cortical stimulation, the quadrilateral configurations with four current sources and one return illustrated relatively even spatial distribution of reflectance changes, indicating specificity improvement with multi-electrode configurations. Besides, to keep the total charge delivered equally in both configurations, the current intensity applied for each electrode in four sources–one return arrangement was reduced to a quarter the intensity delivered in one-source–four returns condition. The stronger reflectance changes with much smaller current intensity delivered from each electrode may also provide a way of thought in the safety of electrical stimulations.

Clinical studies in DBS have shown that by rapidly alternating 2 stimulation configurations, interleaved stimulation could help improve modulation efficacy without eliciting adverse effects [48]. This new approach achieves individualized stimulation current shaping, by variating the stimulation site and electric signals, thus enabling specific therapeutic purposes with fewer side effects related to current spreading to nearby areas. In our study, a simplified interleaved stimulation configuration mode was used. The two stimulation configurations executed were identical, delivering interleaved through two pairs of electrodes. Compared to simultaneous stimulation with the same two pairs of electrodes, interleaved stimulation results in significant drops in both response intensity and spread area. Furthermore, we also monitored cortical responses to bipolar stimulation with the same amount of charge delivered. The results showed that bipolar stimulation evoked significantly larger response intensity as well as spread areas. One possible explanation is that to keep the charge consistent, pulse numbers in bipolar stimulation mode were doubled, leaving the stimulation time consequently prolonged, and therefore, accumulative effects appeared. Nevertheless, the distribution of spread areas with changes more than 3‰, 5‰, 7‰ was observed to be uniform, indicating a controllable current spread within a small region.

## Conclusions

Motor cortex stimulation is a promising therapeutic approach with relatively high spatial and temporal resolution and less invasiveness. Our current study has investigated several stimulation configurations of this approach, with quantitative analysis of both response intensity and spread area, providing preliminary experimental support for parameter and electrode configuration optimizations. More systematic studies and computational/experimental comparisons might be required for detailed understanding and before its utilization as an alternative to conventional stimulation methods.

## Materials and methods

### Subjects and surgical procedures

Six adult male Sprague–Dawley rats weighing between 300 to 350 g were used. The rat was anesthetized with 20% urethane (i.p., 0.7 ml/100 g). After anesthesia, the animal was positioned on a stereotaxic apparatus. A cranial window (5 mm in diameter) was performed over the right primary motor cortex (M1 area), centered at 0.5 mm anterior, 3.5 mm lateral to the bregma according to the Paxinos and Watson brain atlas. The dura was removed, and a 32-channel ECoG electrode array was placed on the cortex. The cranial window was then covered with a coverslip (5 mm in diameter) and fixed to the skull around the cranial window with quick-drying glue. A stainless-steel screw was implanted into the contralateral occipital bone near the lambda, used as a return electrode.

### Electrical stimulation protocol

**Fig. 7:**
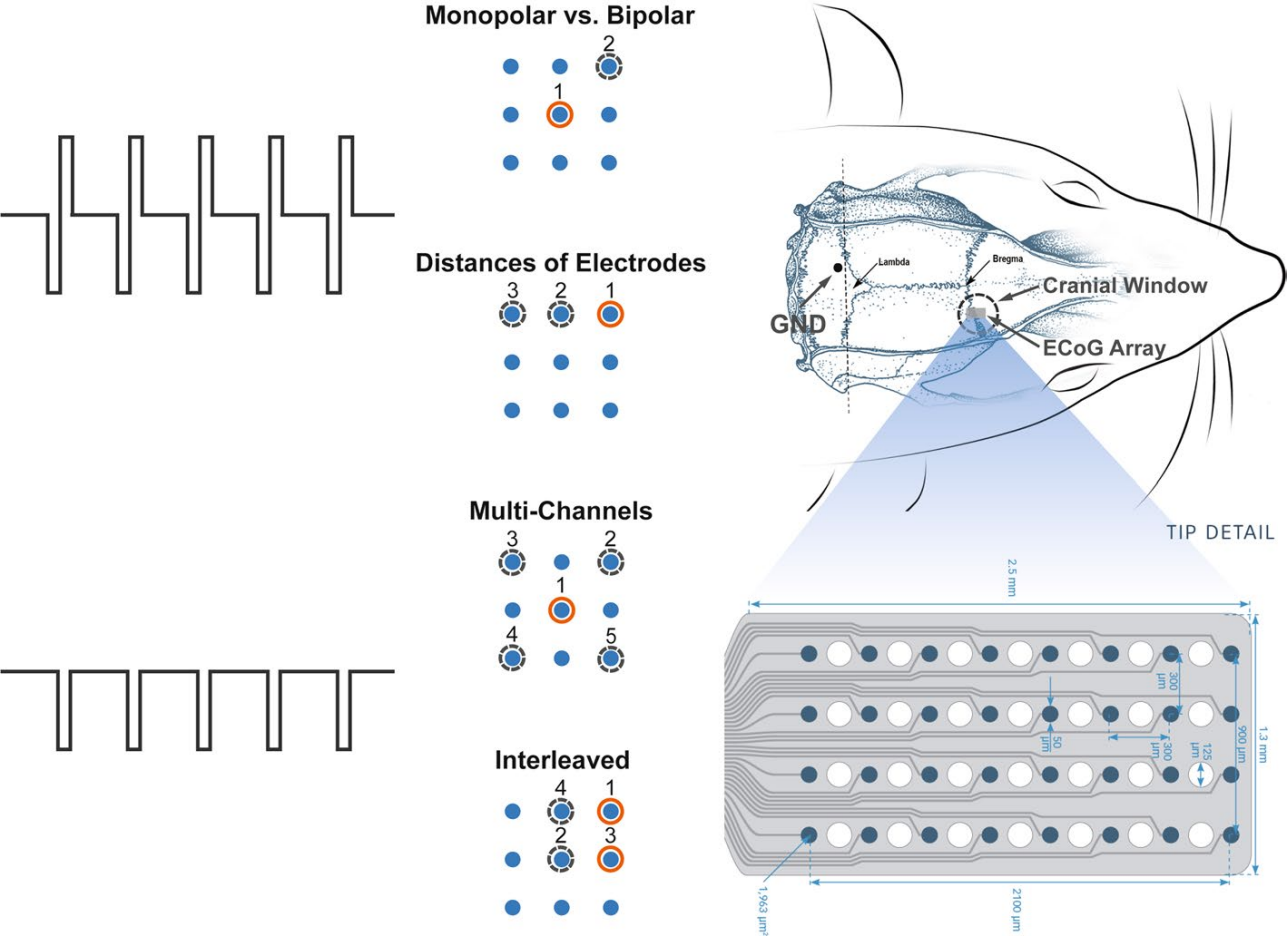
Experimental paradigms. The optical window was opened over the primary motor cortex. Electrical stimulation was delivered through an ECoG array. In conditions of monopolar vs. bipolar stimulation and electrode spacing, charge-balanced biphasic square pulses were applied with cathodic (negative) phase first. In conditions of multi-channel configurations and interleaved stimulation, cathodic pulses were applied

The electrical stimulation was applied through the NeuroNexus ECoG probe (E32-300-20-50, NeuroNexus Technologies, Ann Arbor, Michigan, USA). The electrode array has 32 channels, the size of which is 1.3 mm × 2.5 mm. The electrode contacts (50 μm in diameter) were arranged in a 4 × 8 grid with 300 μm spacing. Stimulation trains were generated by Master-8 pulse stimulator (A.M.P.I., Jerusalem, Israel) and ISO-Flex optical stimulus isolators (A.M.P.I., Jerusalem, Israel). As illustrated in Fig. 7, four configurations/conditions were studied in the current study: (1) comparison between monopolar and bipolar stimulation; (2) impact of distances between electrodes; (3) effects of multi-channel configurations; and (4) effects of interleaved stimulation.

The monopolar stimulations used a cranial screw near the lambda as the return electrode (GND). In addition, the bipolar stimulation used a pair of electrode contacts neighboring on a diagonal to form the circuit (Fig. 7). To measure the responses to parameter changes, charge-balanced biphasic square pulses were applied with cathodic (negative) phase first, each pulse lasting 1 ms, with fixed stimulation duration of 0.1 s. The range of parameters was chosen according to previous reports of other groups and ours [45, 49–52]. The current intensity was tested from 20 to 100 μA with a fixed frequency of 100 Hz, and frequency ranged from 10 to 200 Hz with a fixed current intensity of 60 μA. The impact of electrode distances was tested with bipolar stimulation, with the return electrode shifting from the neighboring contact to a further one. In this condition, electrode distances applied were 300 and 600 μm. Multi-electrode stimulations were applied with quadrilateral configurations, designating one current source/return electrode and four surrounding return/source electrodes accordingly. In this condition, cathodic pulses lasting 1 ms were applied simultaneously through source electrodes at the frequency of 100 Hz, and the stimulation duration lasted 0.03 s for each stimulation trial. To keep the total charge delivered equally, the current intensity was set at 60 μA in one source–four returns configuration while reducing to 15 μA for each source in the four sources–one return configuration. Interleaved stimulations were applied through two pairs of crossed electrode contacts. Cathodic pulses were delivered through the two pairs of electrodes interlaced with a delay of 1 ms. The stimulation parameter was fixed with a current intensity of 60 μA at the frequency of 100 Hz. The cortical responses of this configuration were compared with responses to two-paired simultaneous stimulations.

### Optical imaging of intrinsic signals

Optical imaging of intrinsic signals was performed using Imager 3001 system (Optical Imaging Ltd., Rehovot, Israel) connected to a CCD camera. Before each experiment, the cortex was illuminated with green light (525 nm) for focus adjustment and blood vessel maps obtainment. Functional imaging was then performed under red light (625 nm). Images were recorded at the rate of 10 Hz. Each trial lasted 15 s (150 frames per trial), and the initial 500 ms (5 frames) before electrical stimulation onset was obtained for baseline acquisition. The inter-trial interval was set at 25 s. Every stimulation configuration was repeated 10 times, with each repeated trial included an electrical stimulation condition and a blank condition during which no stimulation was performed, and the two conditions were presented randomly in one trial.

### Data analysis

Image analysis was performed with MATLAB 2016a (MathWorks Inc., Natick, Massachusetts, USA.). All image data were filtered using a Gaussian low pass filter to remove high-frequency noises. To improve the signal-to-noise ratio, 10 repeated trials in the same stimulation configuration were averaged frame by frame, and the blank conditions were subtracted from stimulation conditions. Trials with unstable “blank” conditions were excluded from further analysis.

To examine the stimulation-evoked changes in reflectance compared to baseline, (1) local reflectance changes, and (2) spatial spread of the reflectance changes were considered in the current study. To quantify the local reflectance changes, regions of interest (ROIs, ~ 100 μm in diameter) were selected. The reflectance changes of ROIs were calculated as the average changes of all pixels in the regions and normalized as dR/R(%) = (R_f_–R_b_)/R_b_ × 100%, where R_f_ represents the reflectance value in each imaged frame after stimulation onset, and R_b_ is the averaged reflectance value over the initial baseline frames (first 5 frames). The maximum value of the reflectance change (MRC) was observed, and the time course of the changes was then visualized by plotting dR/R values as a function of time. To determine the spatial spread of the reflectance changes, areas of the image having reflectance changes larger than 3 times the mean square deviation were labeled with red lines as areas with significant changes (ASC) for demonstration in Figs. 1, 2, 3, 4, 5and6. To further estimate the spatial distribution quantitatively, areas of the image having reflectance changes (ARC) more than 3‰, 4‰, 5‰ were also calculated for intergroup comparisons.

### Statistical analysis

Data of the normalized reflectance changes are shown as mean ± standard deviation. Paired student *t* test was applied to each dR/R value to determine if the reflectance changes were significant after stimulations compared to blank trials. Repeated measures analysis of variance (ANOVA) was used to determine statistical significance. Multiple comparisons were performed to compare the response differences among different stimulation conditions using *t* test with Bonferroni adjustment. A *p* value less than 0.05 was viewed as statistically significant.

## Data Availability

The data sets generated and/or analyzed during the current study are available from the corresponding author on reasonable request.

## Abbreviations

DBS: Deep brain stimulation
TMS: Transcranial magnetic stimulation
tDCS: Transcranial direct current stimulation
BMI: Brain–machine interface
ILS: Interleaved stimulation
ROI: Region of interest
MRC: The maximum value of the reflectance change
ASC: Area with significant changes
OI: Intrinsic signal optical imaging
VSD: Voltage-sensitive dye imaging
CBV: Cerebral blood volume
ANOVA: Analysis of variance
