# Correction: Impacts of stimulus parameters and configurations on motor cortex direct electrical stimulation using intrinsic optical imaging: a pilot study

**DOI:** 10.1186/s12938-022-01040-4

**Published:** 2022-09-29

**Authors:** Long Liu, Jiacheng Zhang, Jie Sun, Kedi Xu

**Affiliations:** 1 Zhejiang Provincial Key Laboratory of Cardio‑Cerebral Vascular Detection Technology and Medicinal Effectiveness Appraisal,, Key Laboratory of Biomedical Engineering of Education Ministry, Department of Biomedical Engineering, Zhejiang University, Room 511, Zhou Yiqing Building, 38 Zheda Road, Hangzhou, 310027 China; 2 Department of Neurosurgery, Ningbo City First Hospital, No. 59 Liuting Street, Ningbo, 315010 Zhejiang China; 3 Qiushi Academy for Advanced Studies (QAAS), Zhejiang University, Hangzhou, 310027 China; 4 Zhejiang Lab, Hangzhou, 311100 China

## Correction: BioMedical Engineering OnLine (2022) 21:58 https://doi.org/10.1186/s12938-022-01026-2

Following the publication of the original article [1], the affiliation 1 and 2 need to be swapped and the affiliations should read as follow:

^1^Zhejiang Provincial Key Laboratory of Cardio‑Cerebral Vascular Detection Technology and Medicinal Effectiveness Appraisal, Key Laboratory of Biomedical Engineering of Education Ministry, Department of Biomedical Engineering, Zhejiang University, Room 511, Zhou Yiqing Building, 38 Zheda Road, Hangzhou 310027, China.

^2^Department of Neurosurgery, Ningbo City First Hospital, No. 59 Liuting Street, Ningbo 315010, Zhejiang, China

^3^Qiushi Academy for Advanced Studies (QAAS), Zhejiang University, Hangzhou 310027, China.

^4^Zhejiang Lab, Hangzhou 311100, China.

The original article has been corrected.
